# Unrecognized Primary Hypoparathyroidism with Severe Hypocalcemia in the Presence of COVID-19 Infection

**DOI:** 10.15388/Amed.2021.29.1.9

**Published:** 2022-03-15

**Authors:** Domas Grigoravičius, Laura Šiaulienė, Žydrūnė Visockienė

**Affiliations:** Institute of Clinical Medicine, Faculty of Medicine, Vilnius University, LT-03101, Vilnius, Lithuania; Institute of Clinical Medicine, Faculty of Medicine, Vilnius University, LT-03101, Vilnius, Lithuania; Vilnius University Hospital, Santaros Klinikos, Santariškių Str. 2, LT-08661 Vilnius, Lithuania; Institute of Clinical Medicine, Faculty of Medicine, Vilnius University, LT-03101, Vilnius, Lithuania; Vilnius University Hospital, Santaros Klinikos, Santariškių Str. 2, LT-08661 Vilnius, Lithuania

**Keywords:** primary hypoparathyroidism, hypocalcemia, COVID-19

## Abstract

Primary hypoparathyroidism (PHPT) is a rare disease most commonly caused by surgical parathyroid glands destruction or genetic disorders. PHPT manifestation varies from subclinical to acute or even lethal symptoms. In atypical presentation the signs of hypocalcemia could be missed, and asymptotic chronic hypocalcemia could manifest only in the presence of exacerbated comorbidities, infections, hypomagnesemia or certain medications. We present a case of PHPT with severe hypocalcemia manifesting as seizures and delirium in a presence of COVID-19 infection.

## Santrauka.

Pirminis hipoparatiroidizmas (PHPT) yra reta liga, kurią dažniausiai sukelia chirurginė prieskydinių liaukų destrukcija ar genetinės ligos. Klinikinė PHPT eiga gali varijuoti nuo subklinikinės iki ūmaus ar net mirtino pasireiškimo. Esant atipiniam PHPT pasireiškimui tipiniai hipokalcemijos požymiai gali būti nepastebėti. Be to, asimptominė lėtinė hipokalcemija gali ir nepasireikšti nesant hipokalcemiją skatinančių būklių, tokių kaip infekcijos, hipomagnezemija, tam tikrų vaistų vartojimas. Mes aprašome PHPT atvejį, pasireiškusį sunkia hipokalcemija, traukuliais ir delyru, kartu esant ir nustatytai COVID-19 infekcijai.

## Introduction

Primary hypoparathyroidism (PHPT) is a rare disorder caused by the destruction of the parathyroid glands, abnormal parathyroid gland development, altered regulation of parathyroid hormone (PTH) production, or impaired PTH action [1,2]. The prevalence of this disease varies from 24 per 100 000 in Denmark to 37 per 100 000 people in the United States [3]. More than two-thirds of all patients diagnosed with PHPT are female, and approximately 75% are 45 years and older [3,4]. In about 75 % of cases, PHPT is caused by iatrogenic parathyroid glands destruction during neck surgery [1,5]. Other acquired causes include autoimmune diseases, metastatic disease, hemochromatosis, or Wilson’s disease [4,5]. Type I, III, and IV autoimmune polyendocrinopathy syndromes are clinically relevant, as these disorders are the most frequent form of idiopathic hypoparathyroidism [6]. Most non-surgical cases of PHPT are inherited and can be observed in certain genetic disorders: DiGeorge syndrome, Kenny–Caffey syndrome, Sanjad–Sakati syndrome, Dubowitz syndrome, Bartter syndrome, and Kearns–Sayre syndrome, to name a few [1,2]. Hypoparathyroidism may be associated with different clinical manifestations, ranging from few if any classical symptoms in mild or slowly progressing hypocalcemia to life-threatening sometimes atypical seizures, refractory heart failure, or laryngospasm in severe cases [7]. Long-standing unrecognized hypocalcemia might also cause damage to different organs and systems [7]. Moreover, according to the recent literature, hypocalcemia is associated with the clinical outcomes of COVID-19 infection and can be a prognostic tool for disease severity [8–11].

We present an unusual unrecognized case of primary hypoparathyroidism and hypocalcemia, which manifested as symptomatic seizure and delirium in the presence of COVID-19 infection.

## Case report

**Figure 1.**
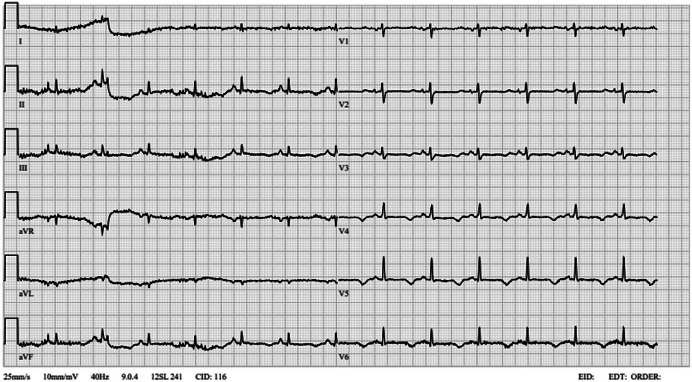
The patient’s initial ECG shows prolonged QT interval and inverted T waves in all leads except V1.

A 39-year-old male was admitted to Vilnius University Hospital Santaros Klinikos Emergency Care Unit (ECU) due to a new-onset of a generalized tonic-clonic seizure. Initial examination showed signs of delirium: aggression, confusion, disorientation in time and place, and inadequate communication. During the physical examination, moderate brain injury (Glasgow Coma Scale (GCS) – 12 points and National Early Warning Score (NEWS) – 2 points), positive Kernig’s sign, pathologic Babinski’s reflexes on both sides, and resting limbs tremor were found. His body temperature was 37.7°C, heart rate (HR) – 81 bpm, blood pressure (BP) – 123/81 mmHg, respiration rate (RR) – 13 breath/min, a saturation of 92.0% on supplemental oxygen. It was known that patient is COVID-19 positive and was self-isolating for three days before admission to ECU. No other clinical information on preexisting medical conditions was established. Biochemical blood analysis showed hypokalemia, increased inflammatory markers, and renal failure (Table 1). ECG analysis revealed prolonged QT interval – QT_C_ 657 ms (normal: 350–440 ms) and inverted T wave in all leads (Figure 1).

**Figure 2.**
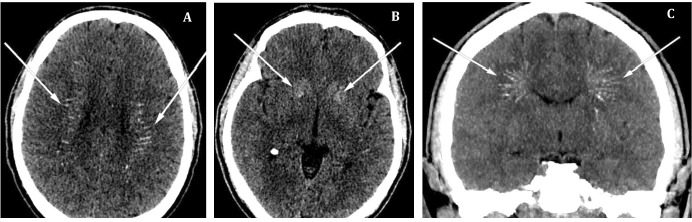
Patient brain CT scan. A, B – transverse plane images; C – coronal plane image. White arrows show intracerebral calcifications.

A preliminary diagnosis of acute COVID-19 associated hemorrhagic necrotizing encephalopathy was made, and specific examination was performed, considering medical history, physical examination, and laboratory findings along with the patient testing positive for COVID-19 infection. Cerebrospinal fluid analysis revealed cytosis and increased protein levels (Table 1). Brain computed tomography (CT) scan showed hyperdense zones bilaterally in basal ganglia, periventricular region, and white matter (Figure 2). Immediate infusion therapy, haloperidol, mannitol, methylprednisolone, vitamin B complex, and ascorbic acid were administered for treatment.

**Figure 3.**
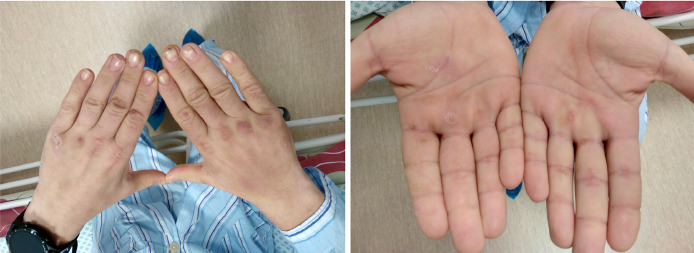
Patient’s hands. Dry skin and onycholysis can be seen.

Due to the remaining mental status deficit and clinically ineffective treatment, the patient’s brain CT scan was revalued, and Fahr’s syndrome – calcifications in *corona radiata* and basal ganglia were identified (Figure 2). Moreover, careful skin and appendages examination revealed dry, yellowish hand and body skin, hyperpigmented palm lines, and onycholysis (Figure 3). Further biochemical analysis revealed severe hypocalcemia, hyperphosphatemia, hypomagnesemia, decreased parathyroid hormone (PTH), and vitamin D levels (Table 2). The level of calcium in the urine was normal (0,65 mmol/l). Neck ultrasound revealed neither thyroid nor parathyroid glands morphologic changes. Abdominal ultrasound showed small amounts of fluid around the liver. Additional medical history revealed that the first-ever seizure the patient had two years ago and was tested for epilepsy. Although the diagnosis was not confirmed, he was preventively using carbamazepine 600 mg/d. The patient also had bilateral cataract extraction and intraocular lens insertion at about the same time. Based on new clinical information, biochemical and instrumental examination, diagnosis of primary hypoparathyroidism with severe hypocalcemia was established. Seizures were classified as symptomatic due to hypocalcemia. Electrolyte disbalance was corrected with calcium, magnesium, and potassium supplements infusions and oral alfacalcidol.

**Figure 4.**
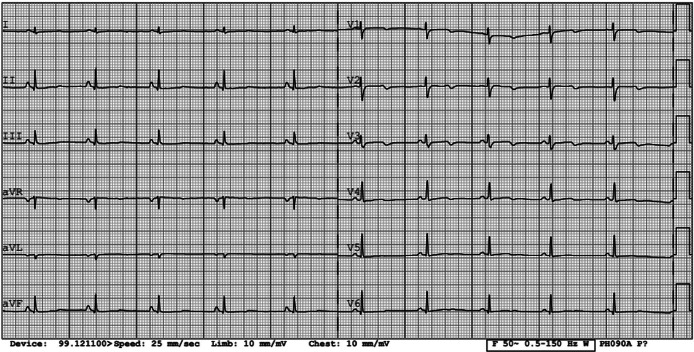
Patient’s ECG after electrolytes correction showing returned to normal QT interval and remained T wave inversion in V2–V4 leads.

**Figure 5.**
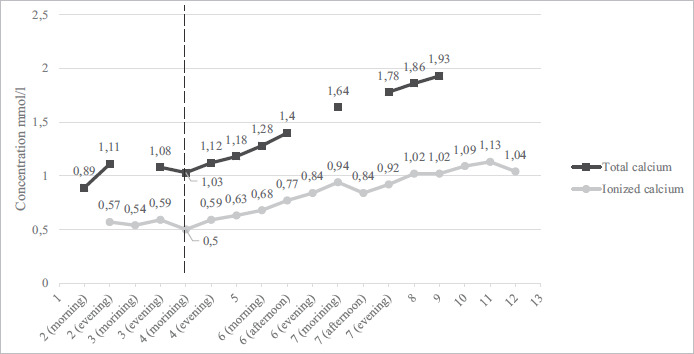
Total and ionized calcium concentration changes during patient hospitalization. Empty parts represent hospitalization days when a test was not performed. The dashed line shows the introduction of intravenous calcium gluconate, oral calcium carbonate, and vitamin D supplementation.

**Figure 6.**
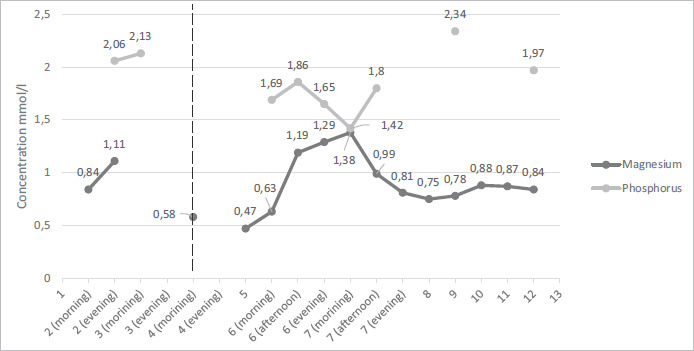
Magnesium and phosphorus concentration changes during patient hospitalization. Empty parts represent hospitalization days when a test was not performed. The dashed line shows the introduction of intravenous magnesium supplementation.

After treatment correction, the patient’s condition improved significantly, and adequate communication was restored. Vital signs were normal with HR 68 bpm, BP 90/60 mmHg, RR 16 breath/min, and saturation of 98% on room air. Repeated ECG showed a return to normal QT interval, while T wave remained inverted in V2-V4 leads (Figure 4). Additionally, morning cortisol, TSH, and FT4 levels were also within normal limits. Patients’ magnesium, calcium, and phosphate concentrations were monitored and kept relatively stable with the prescribed treatment for 15 days while at the hospital (Figure 5 and 6).

The patient was discharged from the hospital with the recommendation to continue calcium (500 mg three times a day), magnesium, and active vitamin D supplements (1,0 µg one time a day). It was recommended to periodically check for electrolytes (calcium, phosphorus, and magnesium) and measure calciuria for treatment correction once a month. Repeated endocrinologist consultation was recommended after three months. The patient was also referred to a genetic consultation.

**Table 1.** Serum biochemistry and liquor analysis on admission day at Emergency Care unit.

Biochemical test	Laboratory value	Normal range
Potassium (mmol/l)	3.0	3.8–5.3
Ferritin (µg/l)	817.69	20–300
Interleukin-6 (ng/l)	16.2	<5.9
C reactive protein (mg/l)	8.24	≤5
Creatinine (µmol/l)	126	64–104
Glomerular filtration rate (mL/min/1.73 m^2^)	47 restored to 92 at discharge	>90
Cytosis (liquor) (/μl)	1	0
Protein level (liquor) (g/l)	0.453	0.15–0.45

**Table 2.** Serum biochemistry day 2.

Biochemical test	Laboratory value	Normal range
Vitamin D (nmol/l)	37.8	75–100
Total calcium (mmol/l)	1.1	2.10–2.55
Ionized calcium (mmol/l)	0.57	1.05–1.30
Magnesium (mmol/l)	0.58	0.65–1.05
Phosphorus (mmol/l)	2.13	0.74–1.52
Parathyroid hormone (pmol/l)	0.33	1.57–7.19
Thyroid stimulating hormone (mU/l)	1.008	0.4–4.0
Free thyroxine (pmol/l)	14.67	9.0–19.0
Morning cortisol (nmol/l)	504	101–536
Adrenocorticotropic hormone (ng/l)	12,3	<46

## Discussion

PTH deprivation usually affects electrolytes homeostasis, causing hypocalcemia and hyperphosphatemia [1]. Generally, hypocalcemia is one of the most common electrolyte disorders that need careful attention [12]. However, the absence of specific clinical signs and explicit etiologic factors can make hypocalcemia a diagnostic challenge [1,12]. The most common manifestation of the disease is neurological and psychiatric symptoms, such as paresthesia, muscle cramps, tetany, anxiety, and depression [1]. However, very often, as it was in our case, these unspecific symptoms remain unreported and undiagnosed. Typical signs of hypocalcemia Trousseau and Chvostek are neither very sensitive nor specific and can be positive in up to 25% of healthy individuals and absent in one-third of patients with hypocalcemia [12,13].

In chronic hypocalcemia, dermatological manifestations, such as dry skin, onycholysis, coarse, thin hair, and pustular psoriasis, are common [1,7]. With further disease progression, bilateral cataracts develop, and musculoskeletal system damage manifests as myopathy and spondyloarthropathy [1]. Our patient presented with onycholysis and dry hand skin as well as bilateral cataracts at a young age. This could be an alert sign for suspicion of hypocalcemia and further investigation. Although rare, cardiovascular system involvement is also seen as arrhythmias and hypocalcemia-induced cardiomyopathy [1,7]. Our patient had typical ECG changes caused by hypocalcemia, which disappeared after the correction of calcium concentration.

Hypoparathyroidism may also be associated with renal failure. According to the literature, renal impairment in most hypoparathyroidism cases is caused by suboptimal treatment with calcium and vitamin D supplements [14,15]. Chronic kidney disease, nephrolithiasis, and renal damage are associated with episodic hypercalcemia, hyperphosphatemia, and vitamin D intoxication [15]. Our patient initially presented with low eGFR before any of the mentioned treatment was provided, which was restored later during the treatment (Table 1). The probable cause of renal impairment, in this case, was dehydration, whereas the patient had a COVID-19 infection [16].

With the progression of the disease and severe calcium depletion, acute life-threatening symptoms, such as delirium or seizures, may occur [7]. According to the literature, delirium is one of the most common acute psychiatric manifestations in PHPT [7]. In contrast, seizures rarely occur in only 4–8 % of cases and can be caused either by acute hypocalcemia or intracerebral calcifications [1,17]. However, seizures and delirium are usually associated with a fast decrease in serum calcium concentration and typically present in post-parathyroidectomy hypoparathyroidism [7]. In other cases, diagnostic difficulties appear – *Li et al.* report hypoparathyroidism in China population are misdiagnosed as epilepsy in 17.55% of total hypoparathyroidism cases [18]. Therefore, due to acute and aggravated presentation, a broad spectrum of delirium and seizures, differential diagnostics, and no anamnestic data on recent neck surgery, pathognomonic signs of hypocalcemia were missed in our patient biochemical work-up of hypocalcemia was delayed. Re-evaluation of brain CT scan was a turning point in this patient differential diagnosis showing cerebral calcification. Intracranial calcifications are present in 52–74% of hypoparathyroidism cases, while basal ganglia calcifications in our patients are pathognomonic to hypoparathyroidism [1,19]. Several case reports also emphasized the importance of radiologic investigation in hypoparathyroidism, where PHPT manifested atypically or was misdiagnosed [17,20,21].

Our patient had evidence of chronic hypocalcemia (bilateral cataracts, skin involvement), and certain factors provoked the abrupt change of calcium levels causing acute presentation. The role of COVID-19 infection in the development of acute hypocalcemia is obscure. There is strong evidence supporting a prognostic hypocalcemia level value in disease severity [8]. The latest systematic review and metanalysis suggest hypocalcemia in patients with COVID-19 can be caused by vitamin D deficiency or over secretion of parathyroid hormone [7]. Hypocalcemia is also commonly observed in patients with non-severe disease, which could be interpreted as a characteristic sign of COVID-19 [9]. However, this needs to be supported by larger studies [9]. Carbamazepine can also cause vitamin D deficiency and hypocalcemia if the drug is used regularly, which was not the case in our patient [22]. Arguably, the least impactful factor in this situation is hypomagnesemia. The literature describes parathyroid glands hypofunction in the presence of severe hypomagnesemia. However, in this case, corrected magnesium levels did not facilitate clinical presentation [14]. Overall, in this case, COVID-19 infection could be an aggravating factor that caused acute symptoms, while the clinical significance of hypomagnesemia and carbamazepine are doubtful.

The patient presented with hyperpigmented palm lines, so autoimmune polyendocrinopathy syndromes (APS) had to be excluded. Normal TSH, FT4, ACTH, and cortisol concentrations ruled out the possible thyroid or adrenal glands deficiency in this patient. Additionally, thyroid ultrasound revealed normal thyroid structure without signs of chronic autoimmune thyroiditis.

The etiology of PHPT in this patient still needs to be clarified, so further genetic analysis for familial disease or de novo mutations is planned. Long-term follow-up strategy involves monitoring serum electrolytes, creatinine, urine calcium excretion, and calcium and vitamin D [1]. Also, routine monitoring of bone mineral density, assessment for the development of signs or symptoms of comorbidities, renal imaging if the patient develops symptoms of renal stone disease are planned [23].

## Conclusions

PHPT is a rare endocrine disorder with a very diverse clinical manifestation. The diagnosis of this condition is relatively simple if serum calcium concentration is measured. Thus, serum calcium should be a routine analysis in all patients with typical or atypical signs and symptoms of chronic or severe hypocalcemia. Also, associated diseases (such as cataract at a young age, epilepsy) or radiological signs of basal ganglia calcification, such as Fahr’s syndrome, should guide the clinician towards suspicion of hypocalcemia. Moreover, special attention should be given to COVID-19 associated hypocalcemia in the presence of the current pandemic.
